# Multi-Dimensional Diffusion Tensor Imaging Biomarkers for Cognitive Decline From the Preclinical Stage: A Study of Post-stroke Small Vessel Disease

**DOI:** 10.3389/fneur.2021.687959

**Published:** 2021-07-12

**Authors:** Jing Du, Hong Zhu, Ling Yu, Peiwen Lu, Yage Qiu, Yan Zhou, Wenwei Cao, Dong Lu, Wei Zhao, Jie Yang, Junfeng Sun, Qun Xu

**Affiliations:** ^1^Renji-UNSW CHeBA (Centre for Healthy Brain Ageing of University of New South Wales) Neurocognitive Center, Renji Hospital, Medical School of Shanghai Jiao Tong University, Shanghai, China; ^2^Department of Neurology, Renji Hospital, Medical School of Shanghai Jiao Tong University, Shanghai, China; ^3^Department of Health Management Center, Renji Hospital, Medical School of Shanghai Jiao Tong University, Shanghai, China; ^4^Shanghai Med-X Engineering Research Center, School of Biomedical Engineering, Shanghai Jiao Tong University, Shanghai, China; ^5^Department of Radiology, Renji Hospital, Medical School of Shanghai Jiao Tong University, Shanghai, China

**Keywords:** cerebral small vessel disease, vascular cognitive impairment, diffusion tensor imaging, structural network, dementia

## Abstract

**Objectives:** We aim to investigate whether multi-dimensional diffusion tensor imaging (DTI) measures can sensitively identify different cognitive status of cerebral small vessel disease (CSVD) and to explore the underlying pattern of white matter disruption in CSVD.

**Methods:** Two hundred and two participants were recruited, composed of 99 CSVD patients with mild cognitive impairment (VaMCI) and 60 with no cognitive impairment (NCI) and 43 healthy subjects as normal controls (NC). Full domain neuropsychological tests and diffusion-weighted imaging were performed on each subject. DTI metrics such as fractional anisotropy (FA), mean diffusivity (MD), the skeletonized mean diffusivity (PSMD), and structural brain network measures including network strength, global efficiency (E_Global_), and local efficiency (E_Local_) were calculated. Region of interest (ROI) analysis of 42 white matter tracts was performed to examine the regional anatomical white matter disruption for each group.

**Results:** Significant differences of multiple cognitive test scores across all cognitive domains especially processing and executive function existed among the three groups. DTI measures (FA, MD, and PSMD) showed significant group difference with the cognitive status changing. FA and E_Global_ showed significant correlation with processing speed, executive function, and memory. ROI analysis found that white matter integrity impairment occurred from the preclinical stage of vascular cognitive impairment (VCI) due to CSVD. These lesions in the NCI group mainly involved some longitudinal fibers such as right superior longitudinal fasciculus (SLF-R), right superior fronto-occipital fasciculus (SFO-R), and right uncinate fasciculus (UNC-R), which might be more vulnerable to the cerebrovascular aging and disease process.

**Conclusions:** DTI measures are sensitive neuroimaging markers in detecting the early cognitive impairment and able to differentiate the different cognitive status due to CSVD. Subtle changes of some vulnerable white matter tracts may be observed from the preclinical stage of VCI and have a local to general spreading pattern during the disease progression.

## Introduction

Cerebral small vessel disease (CSVD) is a term referring to the pathological processes that affect small arteries, arterioles, capillaries, and small veins (1). CSVD is the most common cause of vascular cognitive impairment (VCI), accounting for up to 45% of dementia (2) and 25% strokes (3). The prevalence of CSVD advanced with aging; one study reported that CSVD was observed in 3% of 40- to 49-year-old subjects but 18.9% of 70-year-old subjects (4) and almost 100% in people older than 90 years (5). Given that VCI due to CSVD has an insidious onset with a long deteriorating process, yet few effective treatments are available in the late stage, it is crucial to identify cognitive decline from the very beginning for the purpose of prevention and intervention.

Cerebral small vessels cannot be visualized *in vivo* currently (6); a number of structural brain lesions detected by magnetic resonance imaging (MRI) are regarded as pathologic surrogate markers of cerebrovascular diseases (7). Among them, white matter hyperintensity (WMH), lacunes, cerebral microbleeds, and enlarged perivascular spaces are well-established imaging markers according to the Standards for Reporting Vascular Changes on Neuroimaging (STRIVE) criteria (8). Previous studies have demonstrated that these imaging markers are individually correlated with cognition (9–11), but the strength of the associations are sometimes inconsistent (12). Due to the heterogeneous cerebral vascular brain injuries of the disease, a single lesion may not explain all the variance of cognitive symptoms (13). Moreover, CSVD is more regarded as a “whole brain disease” owing to the similar intrinsic microvascular pathologies of different structural lesions (2). Therefore, it comes to be of great significance to find a synthetic index that can reflect the composite effect of the disease.

DTI is offering an advanced method to detect the white matter microstructure dysfunction (14). Fractional anisotropy (FA) and mean diffusivity (MD) are the most recognized DTI measures, which were reported to be correlated significantly with cognitive impairment due to CSVD in previous studies (15, 16). Peak width of the skeletonized mean diffusivity (PSMD) is another DTI measure, which was proposed in 2016 and has been considered as a sensitive marker of detecting CSVD cognitive decline especially processing speed (17). Recently, structural brain network constructed by white matter tractography is becoming another promising tool for estimating cognitive impairment due to CSVD. Some studies on structural network measures in CSVD patients showed their significant relationship with cognitive performance (18), which seems stronger than individually separate structural lesions do (19). Further analysis indicated that network measures may mediate the relationships between structural lesions with the cognitive performance (18), suggesting that they may be more of the downstream composite biomarkers of cognition.

Given the continuous deteriorating course with insidious onset of the disease, the cognitive status of CSVD may vary from no cognitive impairment (NCI) to mild cognitive impairment (VaMCI) (early clinical stage) and then to vascular dementia (late clinical stage). Some previous observations on CSVD patients have revealed that disruption of white matter integrity has taken place during the VaMCI stage (20, 21). However, to our knowledge, NCI patients with CSVD are not well-investigated in studies as an individual group. In this study, we hypothesized that subtle microstructure changes of white matter may have existed in the preclinical stage of CSVD. To test the hypothesis, multi-dimensional biomarkers derived from DTI techniques that have been widely used for detecting the white matter structure changes were applied to evaluate the brain structural changes of CSVD. All recruited participants were divided into three groups according to the cognitive stage, i.e., NCI, VaMCI, along with the normal controls (NC). The correlations between the cognition and different DTI measures were investigated across these groups. Global FA, MD, PSMD, and structural network measures were applied to investigate the structural changes across the whole brain. ROI (region of interest) analysis for 42 white matter tracts were also conducted to show the anatomical information difference in three groups when the disease deteriorates.

## Materials and Methods

### Participants

Two hundred and two participants were included in this study. One hundred and fifty-nine were non-demented CSVD patients recruited from the stroke clinic at the Department of Neurology, Renji Hospital, an affiliated teaching institution of School of Medicine, Shanghai JiaoTong University. Patients were required to complete a battery of neuropsychological tests and brain MRI scans. Characteristic information including age, sex, education, and vascular risk factors (VRFs) such as hypercholesterolemia, diabetes mellitus, hypertension, and smoking and drinking were collected. The recruitment and exclusion criteria were in accordance with those in our previous study (22), briefly as follows: (1) all the patients were between 55 and 85 years old; (2) education over 6 years; (3) at least 1 month after clinical stroke accident; (4) presence of ≥1 subcortical lacunar infarct(s) and WMH on MRI; and (5) modified Rankin score ≤ 3 (23). Patients were excluded as follows: (1) cognitive impairment due to other degenerative reasons such as Alzheimer's disease (AD) and Parkinson's disease; (2) patients with severe depressive symptoms [17-item Hamilton Depression Rating Scale (HDRS) score ≥ 24 (24)]; (3) intracranial and extracranial vascular stenosis ≥ 50%; (4) cerebral embolisms due to cardiac or other reasons; (5) WMH due to non-vascular dysfunction; and (6) cortical and/or cortico-subcortical non-lacunar territorial infarcts or watershed infarcts. We tried not to include any pure AD patients, but the contamination of the effect of AD might not be totally avoided because AD-related biomarkers such as the E4 variant of apolipoprotein E (APOE) genotype, cerebral spinal fluid amyloid beta or tau, or Amyloid PET scans are not available in this study.

Forty-three healthy subjects from Tangqiao community, Pudong New District in Shanghai, China, were enrolled as NC. The following inclusion criteria for NC were applied: (1) education ≥ 6 years; (2) no history of clinical stroke; (3) no history of severe diseases for important organs, including liver, heart, and lung; (4) WMH ≤ 1 and without other obvious structural abnormalities on MRI scans; (5) HDRS (17 items) score <8; (6) test scores of cognitive assessment were within normal range; and (7) no evidence of vascular risk factors such as hypercholesterolemia, diabetes mellitus, hypertension, and smoking.

Written informed consent forms for all participants were obtained before neuropsychological assessment and MRI scans. The study was approved by the Research Ethics Committee of RenJi Hospital, School of Medicine, Shanghai Jiao Tong University, China.

### Neuropsychological Tests and Diagnosis of Cognitive Status

A standardized battery of multi-domain cognitive tests was performed within a week after MRI acquisition by one trained neuropsychologist. Global cognition was evaluated by the Montreal Cognitive Assessment (MoCA) (25). We also included the following cognitive tests in this study: executive function assessed by trail-making test B (TMT-B) and Stroop color-word test C (SCWT-C); processing speed assessed *via* digit symbol substitution test (DSS); language function assessed by verbal fluency test (VFT, 1-min animal naming test) and 30-item Boston naming test (BNT); visual–spatial function assessed by Rey-Osterrieth complex figure test (Rey-O copy); and memory function evaluated by the auditory verbal learning test (AVLT short-term and long-term recall).

The 17-item HDRS was adopted for rating depressive symptoms. Katz basic activities of daily living (BADL) (26) and Lawton and Brody instrumental activities of daily living (IADL) (27) scales were applied to assess patients' living activity independence. The norms used were based on mean scores of each measurement from a sample of typical, elderly community members in Shanghai, China (28). We defined cognitive impairment as a score of 1.5 standard deviations below the normative means on any of neuropsychological tests. Because some of the patients had a degree of disability due to stroke, we carefully determined which part of the disability was due to cognitive impairment and which was due to physical sequelae. All CSVD patients were categorized into two groups, i.e., VaMCI (*n* = 99) and NCI (*n* = 60). VaMCI was a mild vascular cognitive disorder diagnosed according to the criteria for Vascular Behavioral and Cognitive Disorders (VASCOG) (29), which is in line with the Neurocognitive Disorders Work Group of the fifth revision of the Diagnostic and Statistical Manual of Mental Disorders (DSM-5) (30). These VaMCI patients were diagnosed with one or more impaired cognitive domains but remained independent with regard to daily living ability. NCI was diagnosed for those patients whose neuropsychological test scores were all within the normal range.

### MRI Acquisition

Standardized 3D-fast spoiled gradient recalled (SPGR) sequence images, T2-fluid attenuated inversion recovery (FLAIR) sequences, and diffusion tensor imaging (DTI) were obtained by a professional radiologist using a 3.0-T MRI scanner (Signa HDxt; GE HealthCare, Milwaukee, WI, USA). We acquired 3D-fast SPGR images using the following parameters: TR = 6.1 ms, TE = 2.8 ms, TI = 450 ms, slice thickness = 1.0 mm, gap = 0, flip angle = 15°, FOV = 256 × 256 mm^2^, number of slices = 166; T2-FLAIR images were obtained using the following parameters: TE = 150 ms, TR = 9,075 ms, TI = 2,250 ms, FOV = 256 mm^2^, number of slices = 66; DTI sequences were collected using the following parameters: TR = 17,000 ms, TE = 87.5 ms, matrix = 128 × 128, FOV = 256 × 256 mm, NEX = 1, slice thickness = 2 mm, gap = 0, 20 diffusion-weighted scans with a *b*-value of 1,000 s/mm^2^ and b0 = 0. All subjects including the CSVD patients and NC accepted the MRI scans using the same scanner and parameters.

### DTI Measures

Preprocessing of DTI including eddy current correction and distortion correction was carried out. Tract-based spatial statistics (TBSS) analysis (31) was conducted using FSL (http://www.fmrib.ox.ac.uk/fsl) to generate skeletonized FA and MD. Global FA and MD values were computed by averaging the FA/MD values across the whole skeleton. PSMD was calculated with the PSMD tool provided at http://www.psmd-marker.com (17). A total of 42 ROIs (regions of interest) were delineated based on the JHU white matter parcellation atlas (32) to generate the averaged values across white matter ROIs. The abbreviations of these 42 tracts and their corresponding names are listed in Supplementary Table 2.

### Brain Network Construction

The Pipeline for Analyzing Brain Diffusion Images toolkit (PANDA, www.nitrc.org/projects/panda) was applied to preprocess the raw DTI data (33). The automated anatomical labeling (AAL) template was used to define the network nodes (34), resulting in 45 regions for each hemisphere with cerebellar regions excluded. In brief, T1-weighted images were non-linearly registered to the MNI152_T1_Template (MNI152 standard-space T1-weighted average structural template). Then, the transformation matrix was derived from the registration of b0 images to T1 images. The inverse transformations were applied to the AAL atlas to register the AAL images to DTI native-space cortex parcellations for each subject. Then, each ROI was defined as one node.

Fiber assignment by continuous tracking (FACT) algorithm was performed to generate the whole-brain whiter matter fiber reconstruction. The streamline terminated if the fiber turned at an angle > 45° or met a voxel with an FA < 0.2 (35, 36), because the FA values in a voxel over 0.2 or a sharp bending is unlikely to belong to the bundle of interest. Then, the fiber number (FN) between a pair of ROIs was defined as the interregional structural connectivity. Structural brain networks were finally constructed by a structural 90 × 90 FN-weighted matrix for each subject.

A number of global topological measures were calculated with the graph theoretical network analysis toolbox (GRETNA; http://www.nitrc.org/projects/gretna) (37). Diverse sparsity thresholds ranging from 0.1 to 0.3 with an interval of 0.01 was used to calculate the global and nodal topological properties. Global measures were calculated including network strength, global efficiency (E_Gloal_), and local efficiency (E_Local_). We defined the network strength as the overall accumulation of the FN across all the nodes, which could reflect the connective strength more straightforward. Global efficiency is defined as the average inverse shortest path length for all the node pairs. Local efficiency is defined as the average global efficiency of all direct neighbors of that node, which reflects the efficiency of local information transmission. The areas under the curve (AUC) of each metric across all the thresholds were obtained to summarize the topological characteristic of structural brain network.

### Statistical Analysis

All statistical analyses were performed using SPSS 23.0 (version 23.0, Chicago, IL). The Kolmogorov-Smirnov method was adopted to test the normality of all variables. For normal distributed variables, baseline characteristics were described by mean and standard deviation. For variables with skewed distribution, baseline characteristics were described by median and interquartile range. Gender and vascular risk factors were compared by χ^2^ test between different groups, and one-way ANOVA (analysis of variance) was applied to estimate the group difference of cognitive tests and DWI measures for normally distributed data; Kruskal–Wallis analysis was used for data with high skewness. We applied *post-hoc* analysis to identify pairwise group differences. Bonferroni correction was used for multiple comparisons when conducting the ROI analysis. The relationships between cognitive scores and DWI measures within CSVD patients were evaluated with partial correlation analysis after correcting for age, gender, education, and VRFs. A two-tailed *p*-value of < 0.05 was considered statistically significant.

## Results

### Characteristics of Participants

**Figure 1:**
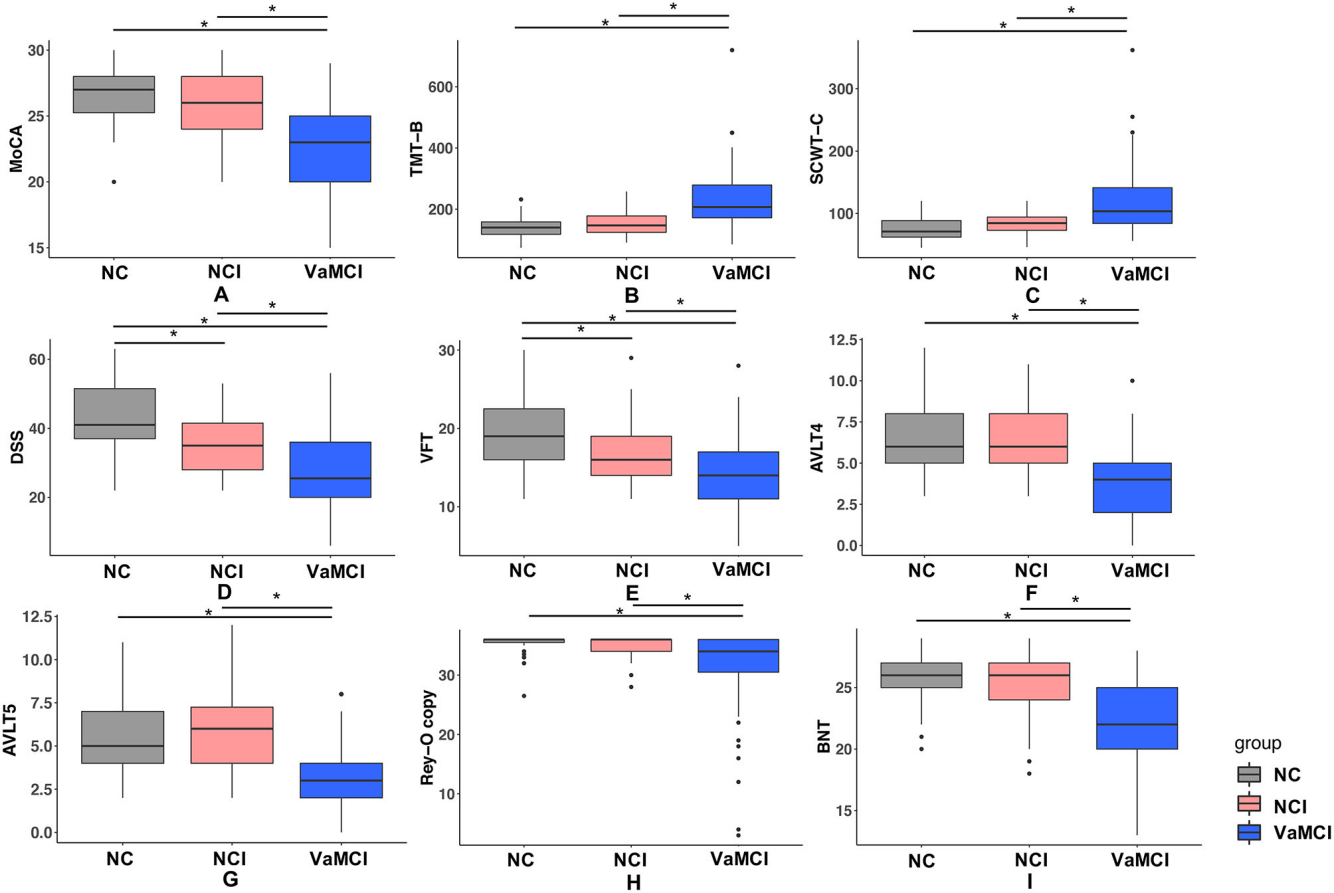
Results of group comparison for different cognitive tests. **(A–I)** One-way ANOVA test was applied to conduct the group comparison of cognitive scores. *p*-value < 0.05 was considered to be statistically significant, * represents *p* < 0.05. VaMCI, CSVD with mild cognitive impairment; NCI, CSVD with no cognitive impairment; NC, normal control; MoCA, Montreal Cognitive Assessment; TMT-B, Trail-Making Tests B; SCWT-C, Stroop color word test C; DSS, digital span substitution test; VFT, verbal fluency test; AVLT-4/5, auditory verbal learning test short and long delayed free recall; BNT, Boston Naming Test; Rey-O copy, Rey-Osterrieth Complex Figure Test (copy).

Baseline characteristics of all participants including demographics and VRFs are listed in Table 1. No significant difference of age and gender was observed among the three groups. *Post-hoc* analysis revealed that education years of the VaMCI group was significantly lower than the NC and NCI group (both *p* < 0.05); no difference of prevalence of VRFs was observed for NCI and VaMCI group. We illustrated the group comparison results of cognitive tests in Figure 1; further descriptive details can be found in Supplementary Table 1. For all cognitive tests, significant difference existed between NC and MC, NCI, and VaMCI, while the difference between NC and NCI was only found for the DSS and VFT.

**Table 1:** Demographic and vascular risk factors description.

	**NC**	**NCI**	**VaMCI**	***p*-value**
	***n* = 43**	***n* = 60**	***n* = 99**	
**Demographic factors**				
Male (%)	31 (72.1)	49 (81.7)	70 (70.7)	0.289
Age, mean (SD)	65.74 (4.806)	65.39 (7.611)	64.17 (7.057)	0.353
Education year, mean (SD)	11.67 (2.741)	11.42 (3.035)	10.26 (2.823)	0.008^a^^,^^b^
**Vascular risk factors**				
Hypercholesterolemia (%)	–	4 (7.0)	11 (12.1)	0.408
Diabetes mellitus (%)	–	21 (39.6)	33 (35.9)	0.722
Hypertension (%)	–	40 (74.1)	72 (77.4)	0.690
Smoking (%)	–	33 (62.3)	40 (44.9)	0.057
Drinking (%)	–	19 (35.2)	25 (28.7)	0.458

*Data represent number (percentage) and mean (standard deviation) for normally distributed data; SD, standard deviation; p-value < 0.05 was considered to be statistically significant; CSVD, cerebral small vessel disease; VaMCI, CSVD with mild cognitive impairment; NCI, CSVD with no cognitive impairment; NC, normal control*;

a *represents significant difference between NC and VaMCI*,

b *represents significant difference between NCI and VaMCI*.

### Group Difference of Neuroimaging Measures

**Figure 2:**
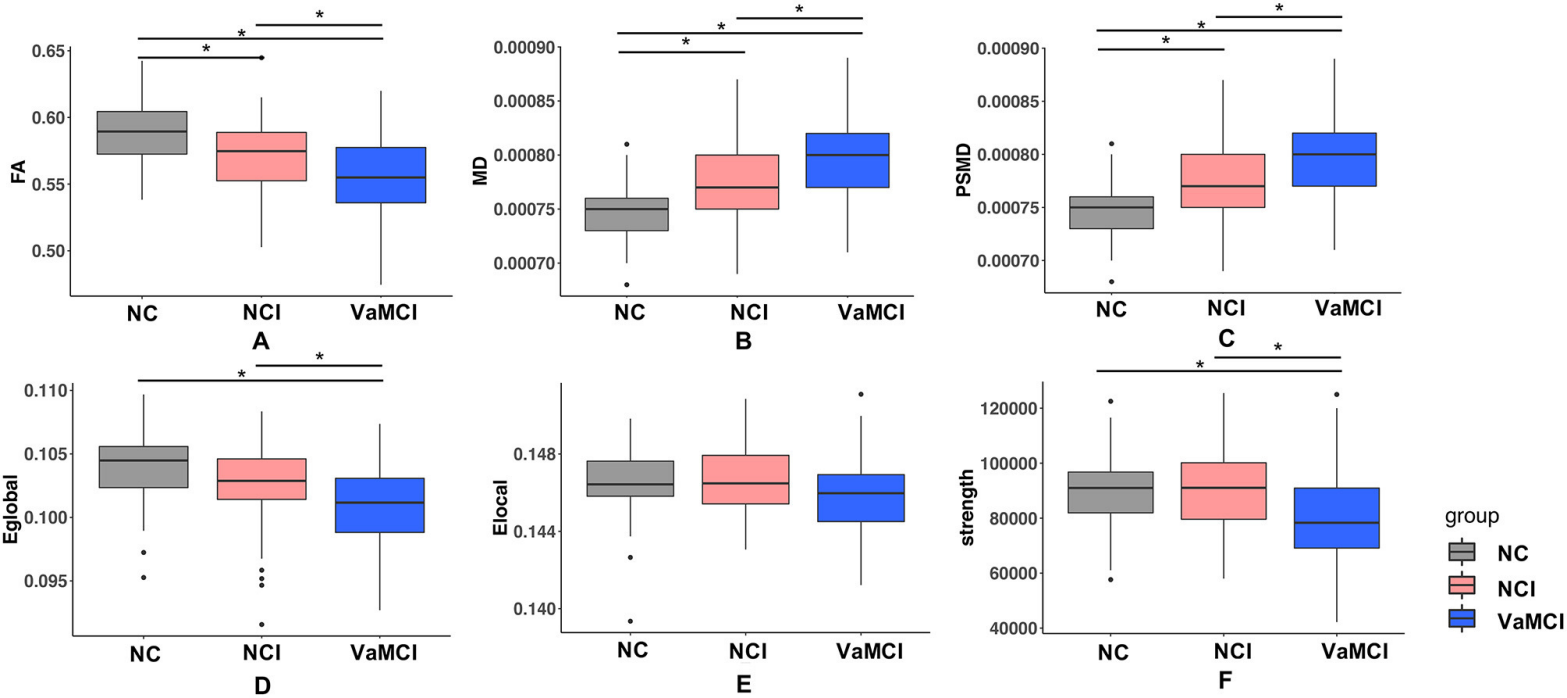
Group difference of DTI measures. **(A–F)** One-way ANOVA test was applied to conduct the group comparison of DTI measures, *p*-value < 0.05 was considered to be statistically significant. * represents *p*-value < 0.05 after Bonferroni correction. VaMCI, CSVD with mild cognitive impairment; NCI, CSVD with no cognitive impairment; NC, normal control; FA, mean fractional anisotropy of the skeleton; MD, mean of mean diffusivity of the skeleton; PSMD, peak width of skeletonized mean diffusivity; E_Global_, global efficiency; E_Local_, local efficiency.

Group comparison results of global DWI measures among the three groups are shown in Figure 2. FA, MD, and PSMD showed significant difference among three groups, but for network measures, both E_Global_ and strength showed significant difference between NC and VaMCI, NCI, and VaMCI, while E_Local_ exhibited no pronounced results after Bonferroni correction.

### Correlations Between Cognitive Scores and DTI Measures Within Non-demented CSVD Patients

The results of correlation analysis between DTI measures and cognition within non-demented CSVD patients are presented in Table 2. After controlling for age, sex, education, and VRFs, correlation analysis showed that E_Global_ was better correlated with cognition covering global cognition, processing speed, executive function, memory, and visual–spatial function (all *p* < 0.05).

**Table 2:** Correlation between DTI measures and cognition within non-demented CSVD patients.

	**FA**		**MD**		**PSMD**		**E**_****Global****_		**E**_****local****_		**Strength**	
	***r***	***p***	***r***	***p***	***r***	***p***	***r***	***p***	***r***	***p***	***r***	***p***
MoCA	0.240	0.011^*^	−0.148	0.122	−0.243	0.011^*^	0.207	0.030^*^	0.233	0.014^*^	0.313	0.001^*^
TMT-B	−0.280	0.002^*^	0.224	0.013^*^	0.210	0.022^*^	−0.218	0.017^*^	−0.218	0.017^*^	−0.143	0.116
SCWT-C	−0.284	0.002^*^	0.258	0.004^*^	0.246	0.007^*^	−0.190	0.037^*^	−0.200	0.029^*^	−0.223	0.014^*^
DSS	0.200	0.023^*^	−0.202	0.022^*^	−0.188	0.033^*^	0.224	0.011^*^	0.239	0.006^*^	0.253	0.004^*^
VFT	0.087	0.329	−0.089	0.315	−0.147	0.096	0.172	0.052	0.101	0.252	0.158	0.073
AVLT4	0.198	0.025^*^	−0.138	0.118	−0.166	0.060	0.224	0.011^*^	0.111	0.209	0.166	0.060
AVLT5	0.208	0.018^*^	−0.146	0.099	−0.127	0.152	0.223	0.011^*^	0.131	0.138	0.146	0.100
Rey-O copy	0.127	0.153	−0.290	0.001^*^	−0.357	<0.001^*^	0.275	0.002^*^	0.196	0.026^*^	0.224	0.011^*^
BNT	0.063	0.480	−0.030	0.734	−0.158	0.074	0.072	0.419	0.064	0.472	0.139	0.116

*FA, mean fractional anisotropy of the skeleton; MD, mean of mean diffusivity of the skeleton; PSMD, peak width of skeletonized mean diffusivity; E_Global_, global efficiency; E_Local_, local efficiency; MoCA, Montreal Cognitive Assessment; TMT-B, Trail-Making Tests B; SCWT-C, Stroop color-word test C; DSS, digital span substitution test; VFT, verbal fluency test; AVLT-4/5, auditory verbal learning test short and long delayed free recall; Rey-O copy, Rey-Osterrieth Complex Figure Test (copy), BNT, Boston Naming Test*;

* *means p < 0.05*.

### ROI Results

**Figure 3:**
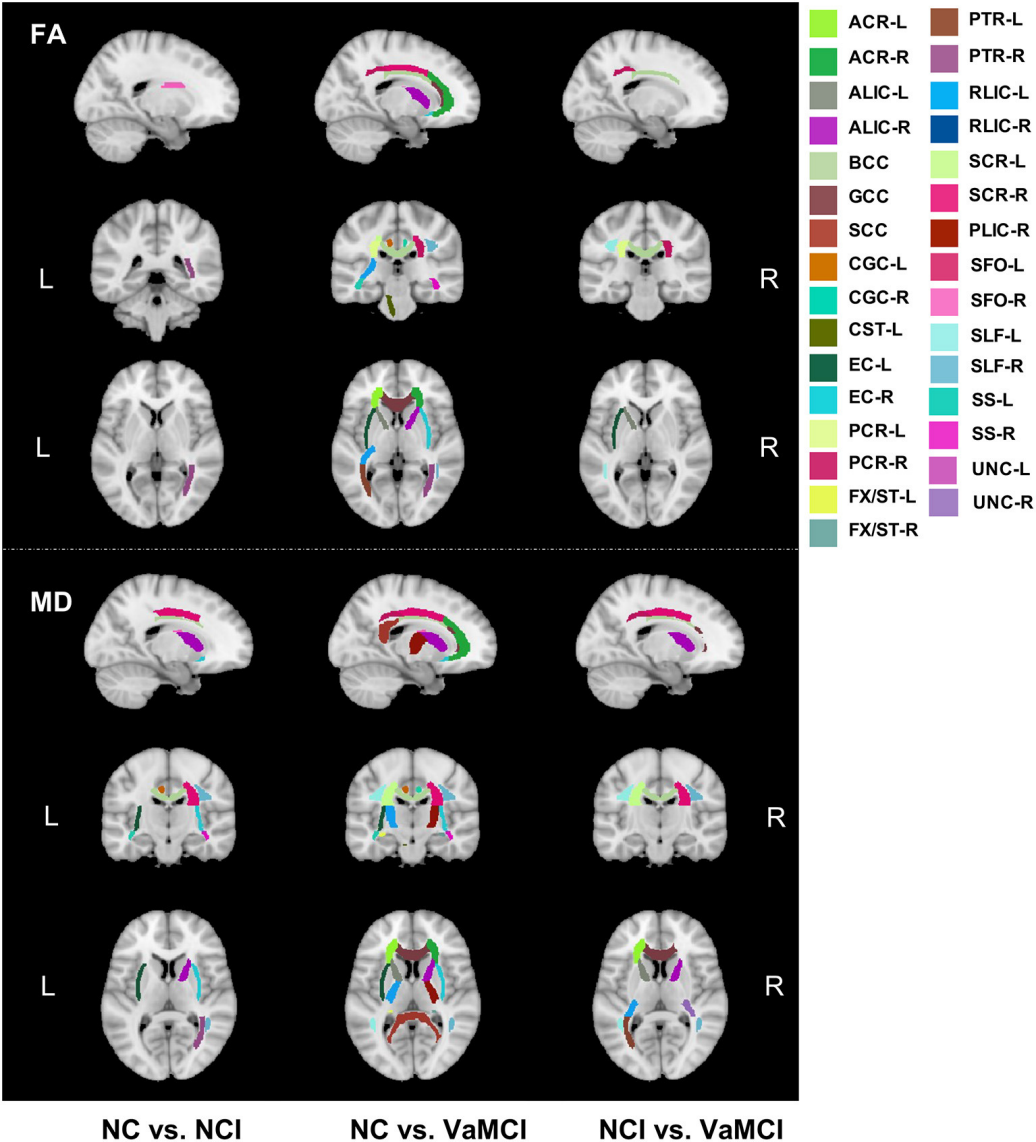
Group comparison results of ROI analysis for 42 white matter tracts. Group comparison of FA and MD on regional white matter tracts. Upper panels show the FA difference between every two groups, and lower panels show the MD results between every two groups. FA, skeletonized fractional anisotropy; MD, skeletonized mean diffusivity, VaMCI, CSVD with mild cognitive impairment; NCI, CSVD with no cognitive impairment; NC, normal control.

Figure 3 illustrated the group comparison results of ROI analysis for 42 tracts. For FA, only right posterior thalamic radiation (PTR-R) and right superior fronto-occipital fasciculus (SFO-R) exhibited a significant difference between the NC and NCI group. Left and right posterior corona radiata (PCR-L/R), body of corpus callosum (BCC), left external capsule (EC-L), and left superior longitudinal fasciculus (SLF-L) show a significant difference between NCI and VaMCI. For the difference between NC and VaMCI, we found the widespread impairment covering more white matter tracts. Further details can be found in Supplementary Table 3. For MD, ALIC-R, BCC, right corona radiata (CR-R), left cingulum cingulate part (CGC-L), EC-L/R, left and right internal capsule (IC-L/R), PTR-R, right superior corona radiata (SCR-R), SFO-R, SLF-R, left and right sagittal stratum (SS-L/R), and right uncinate fasciculus (UNC-R) were found to be significantly different between NC and NCI; as to the difference between NCI and VaMCI, left anterior corona radiata (ACR-L), ALIC-L/R, BCC, CR-L/R, genu of corpus callosum (GCC), IC-L/R, PCR-L/R, PTR-L, left and right retrolenticular part of internal capsule (RLIC-L/R), SCR-L/R, SLF-L/R, and UNC-L showed significant results. In the VaMCI group compared with the NC group, we found the impairment covering almost all white matter tracts except for left and right cingulum (hippocampus) (CGH-L/R), left corticospinal tract (CST-L), fornix (FX) and left inferior fronto-occipital fasciculus (IFO-L), SS-L/R, and UNC-L/R. We also conducted the correlation analysis between cognition and 42 ROI tracts. Most of the significant relationships were observed with TMT-B and SCWT-C. The detailed results are listed in Supplementary Tables 4, 5.

## Discussion

In this study, we included non-demented CSVD patients as well as healthy NC. The results demonstrated that DTI measurements deteriorated progressively from the NC to the preclinical stage of VCI (NCI) and then VaMCI (the early stage). Three main findings were derived from this study. Firstly, DTI measures could distinguish different stages of VCI due to CSVD, even from the preclinical phase. Secondly, FA and E_Global_ showed more widespread correlations with test scores of different cognitive domains including processing speed, executive function, and memory than other DTI measures. Thirdly, ROI analysis revealed that even from the preclinical stage of VCI, some subtle changes of the connection involving the projection and association fibers have taken place. CSVD patients exhibited a gradually generalized white matter integrity disruption with the disease progression.

DTI is an advanced and non-invasive technique for evaluating the white matter integrity, which is usually considered to be highly correlated with the cerebrovascular health. In this study, we applied different DTI measures to explore the relationship between the white matter micro-structural changes and cognition manifestation in CSVD patients. Cognitively intact CSVD patients are those who could be regarded as the potential candidates of VCI who are at the very beginning stage. However, they received little attention in previous studies compared with VaMCI or vascular demented patients. For disease-prevention-oriented purposes, more concerns are needed for these patients. In our study, DSS and VFT were observed impaired in the NCI stage compared to NC. DSS is commonly applied to evaluate the executive function; to our knowledge, VFT is usually used to assess the verbal function (language) or processing speed and executive function (38). Moreover, with the progression of the disease, significant group differences between NCI and MCI were observed across all cognitive domains. Processing speed and executive functions are usually thought primarily to be the most vulnerable cognitive domains in cerebrovascular diseases. Different from AD, CSVD will also affect all major domains of cognitive ability gradually (39). The correlation analysis between DTI measures and cognition within CSVD patients indicated that most DTI measures showed significant correlation with processing speed and executive, but few were correlated with language, memory, and spatial function. Brain network measures especially E_Global_ showed strong correlation with most of the cognitive tests. Currently, CSVD is considered to be a global disease rather than a focal one, since various structural lesions may generate remote effects on structural and functional network connections (40). Previous studies have demonstrated that brain network characters have advantages of explaining cognitive dysfunction than conventional imaging markers (41). Our study demonstrated that global efficiency outperformed other global DTI measures and was correlated with almost all cognitive domains. Graph theory measures were calculated based on the structural network, which have been reported to be able to detect the integration of information across the whole brain (14, 42). Cognitive impairment arose while the white matter circuitry was impaired in CSVD (43). Instead of isolated brain regions, it might be the networks formed by different neural circuits or brain regions that affect the cognitive performance.

In addition to the global DTI measures, we further investigated the tract-based DTI measures to determine the particularly vulnerable tracts during the disease course. The results indicated that increasing tracts were affected during the process of cognitive deterioration. For both FA and MD, SFO-R and PTR-R were impaired in NCI compared to NC, SFO was considered connecting the frontal lobe to ipsilateral parietal lobe for symmetric processing and spatial awareness (44), while PTR was also reported to be part of the pathway that appears most susceptible to aging and risk factors (45). Besides, the tracts involving significant group differences for MD also outnumbered that for FA. For FA, only two tracts were found to be significantly between NC and NCI, whereas 15 tracts were found to be significantly different for MD between NC and NCI. We also found more other association and projection fibers impaired for MD in the NCI group compared to the NC group, which may provide the hint that MD might be more sensitive than FA to monitor the white matter disruption of CSVD. Some previous studies also reported that MD might be more sensitive in detecting mild injury, whereas FA captures more severe injuries (46, 47). However, some studies also reported opposite findings in which FA might be more sensitive for detecting the cognitive changes (48). Long association fibers such as SLF-R, SFO-R, and UNC-R showed a significant MD increase at the NCI stage. These long association fibers were usually thought to be myelinated relatively late and vulnerable to insult and decline in later life (49). The “last in first out” mechanism has been proposed in that brain regions that developed late will be more vulnerable to the aging process (50). Both SLF and SFO are important tracts in the frontal–parietal–subcortical networks, which have been reported to be essential circuits in CSVD (51). Myelin damage was thought to be associated with transmission speed reduction and was reported to be correlated with cognitive decline in CSVD (52). The impairment of corpus callosum, internal capsule, and external capsule in NCI in this study was also observed in a previous study in VaMCI patients with CSVD (20). Furthermore, uncinate fasciculus, which connected the frontal and temporal lobes, was also observed to be impaired in NCI compared to the NC group. MD values for CGC (cingulate gyrus) also increased in NCI. Frontal lobe and CGC were reported to be the essential nodes in the long association pathway in frontal–subcortical neuronal circuits, closely correlated with the executive function in CSVD (53, 54). We also observed the significant difference of MD between NC and NCI in ALIC, which was consistent with previous observations wherein fibers in ALIC develop considerably later in contrast to PLIC (55). Moreover, the correlation analysis between cognition and FA/MD values in regional tracts also demonstrated that processing and executive are the two susceptible domains. To conclude, our results provided evidence to a local to general connectivity dysfunction pattern in the full course development of VCI in CSVD, and at the super preclinical stage without any cognitive impairment, many subtle changes have taken place in some susceptible white matter tracts associated with processing and executive function. Interestingly, we did not find any significant group difference of CGH and fornix between NC and NCI in this study. Previous studies regarding Alzheimer's disease reported that CGH (cingulum hippocampus part) and fornix function are usually susceptible to be impaired in the stage of preclinical AD (56) and were highly correlated with memory function.

Several limitations are worth to be mentioned in this study. Firstly, this is a cross-sectional cohort study, which cannot derive a causative relationship between brain structural changes and cognitive decline. Future multi-centered and longitudinal studies with large samples are needed to investigate the underlying mechanism of occurrence and progression of VCI due to CSVD. Secondly, the FACT streamlining algorithm we used to construct the network has several limitations in tracking the fibers in complex white matter architecture especially the crossing fibers; a better alternative method is to obtain the advanced white matter imaging techniques such as high angular resolution diffusion imaging (HARDI) and diffusion spectrum imaging (DSI), which can provide superior qualitative data in terms of multiple crossing fibers with high spatial orientation. Thirdly, even though we have made every effort to exclude the effect of AD, it could not be totally excluded from those CSVD patients since it is difficult to disentangle accurately whether AD or vascular pathologies are driving the observed effects.

In this study, different DTI measures were applied to investigate CSVD patients with different cognitive statuses. CSVD patients with no significant cognitive impairment have already presented with impaired white matter integrity, suggesting that DTI measures may be regarded as sensitive markers in detecting the insidious onset of VCI. Subtle changes of some specific longitudinal tracts such as SLF-R, SFO-R, and UNC-R involving the frontal–parietal–subcortical network have been captured in the preclinical stage of VCI, which might add more knowledge to the underlying pattern of white matter disruption in CSVD.

## Data Availability Statement

The datasets generated for this study are available on request to the corresponding author.

## Ethics Statement

The studies involving human participants were reviewed and approved by Research Ethics Committee of RenJi Hospital, School of Medicine, Shanghai Jiao Tong University, China. The patients/participants provided their written informed consent to participate in this study.

## Author Contributions

QX, JS, and YZ conceived and designed the experiments. LY, PL, DL, WZ, WC, and YQ performed the experiments. JD and HZ analyzed the data and wrote the paper. QX and JS supervised the study and revised the paper. All authors contributed to the article and approved the submitted version.

## Conflict of Interest

The authors declare that the research was conducted in the absence of any commercial or financial relationships that could be construed as a potential conflict of interest.
