# Supplementary material for: Multi-Dimensional Diffusion Tensor Imaging Biomarkers for Cognitive Decline From the Preclinical Stage: A Study of Post-stroke Small Vessel Disease
Source: Front Neurol. 2021 Jul 12;12:687959. doi: 10.3389/fneur.2021.687959 (PMC8311001; doi:10.3389/fneur.2021.687959)
Supplement: Supplementary file 1 [file Data_Sheet_1.pdf]

Supplementary Material

Supplementary Table 1 Description of different cognitive scores in three groups

	NC (n = 43)	NCI (n = 60)	VaMCI (n = 99)
MoCA, mean (sd)	26.71 (2.21)	26.16 (2.30)	22.38 (3.26)
TMT-B, median (interquartile range)	140 (42)	147 (58)	207 (110)
SCWT-C, median (interquartile range)	71 (27)	84.5 (22)	103.50 (58)
DSS, mean (sd)	43.49 (10.25)	35.32 (8.75)	27.77 (10.73)
VFT, mean (sd)	19.47 (4.42)	16.75 (3.74)	13.93 (4.50)
AVLT-4, mean (sd)	6.42 (2.02)	6.45 (1.87)	3.85 (1.87)
AVLT-5, mean (sd)	5.67 (1.95)	5.98 (2.10)	3.05 (1.97)
Rey-O copy, median (interquartile range)	36 (1.0)	36 (2.0)	34 (6.0)
BNT, mean (sd)	25.98 (2.14)	25.57 (2.65)	21.98 (3.84)

Abbreviations: MoCA, Montreal Cognitive Assessment; TMT-B, Trail-Making Tests B; SCWT-C, Stroop color-word test C; DSS, digital span substitution test; VFT, verbal fluency test; AVLT-4/5, auditory verbal learning test short and long delayed free recall; Rey-O copy, Rey-Osterrieth Complex Figure Test (copy), BNT, Boston Naming Test; VaMCI, CSVD with mild cognitive impairment; NCI, CSVD with no cognitive impairment; NC, normal control; sd, standard deviation.

Supplementary Table 2 Abbreviations and full names for white matter tracts

Abbreviation	Full tract name
ACR (L/R)	Anterior corona radiata
ALIC (L/R)	Anterior limb of internal capsule
BCC	Body of corpus callosum
CGC (L/R)	Cingulum (cingulate gyrus)
CGH (L/R)	Cingulum (hippocampus)
CR (L/R)	Corona radiata
CST (L/R)	Corticospinal tract
EC (L/R)	External capsule
FX	Fornix
FXST (L/R)	Fornix (cres) / Stria terminalis
GCC	Genu of corpus callosum
IC (L/R)	Internal capsule
IFO (L/R)	Inferior fronto-occipital fasciculus
PCR (L/R)	Posterior corona radiata
PLIC (L/R)	Posterior limb of internal capsule
PTR (L/R)	Posterior thalamic radiation
RLIC (L/R)	Retrolenticular part of internal capsule
SCC	Splenium of corpus callosum
SCR (L/R)	Superior corona radiata
SFO (L/R)	Superior fronto-occipital fasciculus
SLF (L/R)	Superior longitudinal fasciculus
SS (L/R)	Sagittal stratum
UNC (L/R)	Uncinate fasciculus

Supplementary Table 3 ANOVA results of 42 white matter tracts among three groups

	FA			MD		
	NC vs. NCI	NC vs. VaMCI	NCI vs. VaMCI	NC vs. NCI	NC vs. VaMCI	NCI vs. VaMCI
ACR-L	0.200496	0.000006 [b]	0.005486	0.00395	1.51E-10 [b]	0.0007 [c]
ACR-R	0.031871	1.2217E-07 [b]	0.004941	0.004964	1.90E-09 [b]	0.003076
ALIC-L	0.308601	0.000003 [b]	0.00121	0.001935	1.67E-11 [b]	0.000368 [c]
ALIC-R	0.00257	1.5856E-08 [b]	0.02498	0.000339 [a]	2.40E-12 [b]	0.000792 [c]
BCC	0.012987	2.2218E-09 [b]	0.00096 [c]	0.000577 [a]	1.95E-12 [b]	0.000368 [c]
CGC-L	0.504055	0.000018 [b]	0.002048	0.0003 [a]	1.99E-11 [b]	0.003623
CGC-R	0.140395	0.000026 [b]	0.028187	0.006687	0.000008 [b]	0.320208
CGH-L	1	1	0.596413	1	0.959233	0.580943
CGH-R	1	1	0.570653	1	0.489579	0.739975
CR-L	0.071853	4.0045E-08 [b]	0.000592 [c]	0.001713	2.06E-12 [b]	0.000093 [c]
CR-R	0.008012	8.8637E-10 [b]	0.000966 [c]	0.000586 [a]	8.44E-13 [b]	0.000198 [c]
CST-L	0.015004	0.000396 [b]	1	0.00954	0.001335	1
CST-R	0.030893	0.017285	1	0.00192	0.000537 [b]	1
EC-L	0.033873	1.3966E-09 [b]	0.000149 [c]	0.00012 [a]	6.36E-12 [b]	0.00447
EC-R	0.006101	1.0664E-08 [b]	0.00765	0.000004 [a]	7.58E-13 [b]	0.026089
FX	0.511515	0.003832	0.159892	0.522667	0.094577	1
FS/ST-L	1	0.018545	0.048184	0.012622	0.000001 [b]	0.065777
FS/ST-R	0.36214	0.003446	0.239482	0.092681	0.000005 [b]	0.014797
GCC	0.30913	0.000006 [b]	0.002241	0.019101	2.49E-09 [b]	0.000592 [c]
IC-L	0.069403	0.000003 [b]	0.017836	0.000375 [a]	8.29E-13 [b]	0.00034 [c]
IC-R	0.016509	0.000003 [b]	0.092308	0.000183 [a]	1.01E-13 [b]	0.000183 [c]
IFO-L	1	0.020942	0.179589	1	0.381889	0.075346
IFO-R	1	0.068385	0.138348	0.314672	0.000298 [b]	0.049765
PCR-L	0.197571	1.2808E-08 [b]	0.000029 [c]	0.012527	2.81E-12 [b]	0.000005 [c]
PCR-R	0.135631	1.2953E-07 [b]	0.000477 [c]	0.016815	2.07E-10 [b]	0.000105 [c]
PLIC-L	0.054241	0.009511	1	0.003386	0.000002 [b]	0.269902
PLIC-R	0.063518	0.002007	0.925084	0.004992	8.81E-08 [b]	0.035928
PTR-L	0.023286	3.0328E-08 [b]	0.002818	0.205067	4.74E-07 [b]	0.00062 [c]

PTR-R	0.000222[a]	2.9295E-11 [b]	0.006247	0.001161 [a]	1.89E-08 [b]	0.057748
RLIC-L	0.196624	0.000066 [b]	0.033501	0.003563	5.17E-11 [b]	0.00037 [c]
RLIC-R	0.523175	0.006247	0.222006	0.007696	5.75E-11 [b]	0.000133 [c]
SCC	1	0.003501	0.04436	0.016679	6.37E-09 [b]	0.001455
SCR-L	0.089957	0.000005 [b]	0.016251	0.002918	1.83E-10 [b]	0.001188 [c]
SCR-R	0.015457	8.9186E-07 [b]	0.045428	0.000232 [a]	1.17E-12 [b]	0.000752 [c]
SFO-L	0.412552	0.008417	0.374633	0.117766	0.000051 [b]	0.05754
SFO-R	0.000157[a]	8.827E-11 [b]	0.01657	0.000276 [a]	4.03E-09 [b]	0.082018
SLF-L	0.018498	3.1971E-09 [b]	0.00075 [c]	0.01191	1.26E-11 [b]	0.00002 [c]
SLF-R	0.001821	3.4649E-08 [b]	0.054379	0.00035 [a]	2.06E-12 [b]	0.00069 [c]
SS-L	0.074971	7.5748E-07 [b]	0.005348	0.000752 [a]	1.08E-10	0.004035
SS-R	0.002284	3.7041E-08 [b]	0.045948	0.000407 [a]	3.17E-09	0.05311
UNC-L	0.037163	1.9044E-07 [b]	0.005474	0.01076	3.93E-11	0.000058 [c]
UNC-R	0.015704	5.0449E-09 [b]	0.001337	0.000629 [a]	1.34E-10	0.005581

Table1: *Abbreviations: CSVD, cerebral small vessel disease; VaMCI, CSVD with mild cognitive impairment; NCI, CSVD with no cognitive impairment; NC, normal control; a represents significant difference between NC and NCI, b represents significant difference between NC and VaMCI, c represents significant difference between NCI and VaMCI. P-value < 0.05 was considered to be statistically significant after Bonferroni correction (original p-value*3*42); FA, mean fractional anisotropy of the skeleton; MD, mean of mean diffusivity of the skeleton.*

Supplementary Table 4 Correlation between tract-based FA and different cognitive scores

	MoCA	TMT-B	SCWT-C	DSS	VFT	AVLT4	AVLT5	Rey-O copy	BNT
ACR_L	0.1327	0.010155	0.025996	0.20935	0.16646	0.13269	0.17065	0.002925	0.73933
ACR_R	0.20403	0.011721	0.017940	0.088	0.13498	0.09201	0.09408	0.014921	0.74313
ALIC_L	0.02682	0.004006	0.005360	0.43683	0.87739	0.004	0.00284	0.003027	0.38912
ALIC_R	0.31832	0.006026	0.014815	0.24806	0.92424	0.0959	0.06042	0.011845	0.86791
BCC	0.01411	**0.000069**	**0.000651**	0.00687	0.09746	**0.00092**	0.00155	0.040242	0.57416
CGC_L	0.00418	**0.001022**	**0.001123**	0.02008	0.04883	0.00183	**0.00035**	0.602039	0.81614
CGC_R	0.01667	0.014400	0.014660	0.06838	0.22001	0.03429	0.01397	0.366664	0.48151
CGH_L	0.69251	0.301027	0.802728	0.87994	0.12224	0.86034	0.42485	0.324765	0.62975
CGH_R	0.63109	0.317041	0.743093	0.75616	0.06439	0.77507	0.4899	0.867728	0.9781
CR_L	0.04867	0.011323	0.005264	0.10047	0.38126	0.0502	0.13012	0.006488	0.56482
CR_R	0.0271	0.019788	0.004339	0.09412	0.17269	0.05588	0.079	0.012604	0.37318
CST_L	0.67391	0.536601	0.466405	0.53103	0.1704	0.35309	0.61482	0.804336	0.04531
CST_R	0.48457	0.465556	0.935533	0.97904	0.69483	0.59791	0.98116	0.858526	0.37016
EC_L	0.0294	**0.000456**	0.007235	0.01107	0.47588	0.01447	0.00931	0.018169	0.3602
EC_R	0.06156	0.005290	0.010151	0.00221	0.38121	0.06998	0.04705	0.057277	0.27749
FX	0.51571	0.091047	0.412636	0.09101	0.11502	0.23402	0.28674	0.749819	0.16552
FXST_L	0.00256	0.207614	0.393516	0.0055	0.28907	0.17303	0.05872	0.382788	0.50963
FXST_R	0.04219	0.044789	0.186788	0.00707	0.7707	0.21267	0.13336	0.904043	0.1122
GCC	0.051	0.007659	**0.001111**	0.06088	0.24546	0.00822	0.0065	0.036984	0.18362
IC_L	0.1505	0.047967	0.015457	0.66651	0.47733	0.02189	0.01938	0.043956	0.99556
IC_R	0.39323	0.253259	0.100639	0.95093	0.48267	0.20908	0.11819	0.106842	0.79542
IFO_L	0.09215	0.035355	0.007078	0.02451	0.23515	0.00122	0.00338	0.064296	0.45449
IFO_R	0.32296	0.573442	0.091334	0.29347	0.30271	0.19199	0.14156	0.374460	0.8107
PCR_L	0.05765	0.018546	0.003793	0.25561	0.50585	0.02248	0.10452	0.068284	0.24072
PCR_R	0.00511	0.028354	0.003018	0.08763	0.39172	0.01569	0.06931	0.045492	0.11328
PLIC_L	0.90132	0.485439	0.604320	0.74187	0.03866	0.4438	0.39735	0.846564	0.3703
PLIC_R	0.91578	0.818762	0.612698	0.27705	0.27435	0.7547	0.65893	0.221963	0.57779
PTR_L	0.15616	**0.000862**	**0.000309**	0.01062	0.23904	0.04592	0.03785	0.236359	0.95292
PTR_R	0.03796	0.055271	0.007478	0.02142	0.23207	0.09133	0.03773	0.310543	0.90136
RLIC_L	0.36028	0.303421	0.014582	0.63055	0.88385	0.06733	0.08084	0.092251	0.79764
RLIC_R	0.2277	0.923598	0.283657	0.58323	0.32511	0.26655	0.11653	0.521651	0.70717
SCC	0.14554	0.023263	0.001706	0.0094	0.07165	0.0234	0.13105	0.030713	0.64072
SCR_L	0.04618	0.097567	0.013912	0.0658	0.99016	0.08909	0.26988	0.142416	0.69655
SCR_R	0.04171	0.255003	0.036557	0.38166	0.35679	0.29266	0.29107	0.236141	0.43244
SFO_L	0.163	0.032696	0.150806	0.28834	0.62454	0.03244	0.00869	0.132215	0.31857
SFO_R	0.05757	0.005622	0.006485	0.02656	0.20859	0.05284	0.01754	0.013962	0.22299
SLF_L	0.0276	0.020519	0.004846	0.03525	0.17299	0.01411	0.05029	0.100799	0.78925
SLF_R	0.07825	0.026924	0.058695	0.01798	0.17485	0.28071	0.58965	0.015344	0.62524
SS_L	0.09298	0.029869	0.005445	0.02858	0.16939	0.11528	0.0842	0.211665	0.985

SS_R	0.10716	0.212342	0.040081	0.08438	0.87726	0.55873	0.20488	0.371347	0.73673
UNC_L	0.16619	0.111637	0.005932	0.07029	0.99578	0.05456	0.20888	0.017441	0.6633
UNC_R	0.11171	0.011652	0.002781	0.02993	0.62007	0.01433	0.03114	0.003892	0.40665

Partial correlation analysis between tract-based FA and different cognitive scores after correcting for age, sex, education and vascular risk factors. Abbreviations: MoCA, Montreal Cognitive Assessment; TMT-B, Trail-Making Tests B; SCWT-C, Stroop color-word test C; DSS, digital span substitution test; VFT, verbal fluency test; AVLT-4/5, auditory verbal learning test short and long delayed free recall; Rey-O copy, Rey-Osterrieth Complex Figure Test (copy), BNT, Boston Naming Test. Bold p values are statistically significant after Bonferroni correction.

Supplementary Table 5 Correlation between tract-based MD and different cognitive scores

	MoCA	TMT-B	SCWT-C	DSS	VFT	AVLT4	AVLT5	Rey-O copy	BNT
ACR_L	0.071807	0.007039	0.025335	0.016196	0.067967	0.009192	0.039781	0.014174	0.543900
ACR_R	0.191674	0.005159	0.009538	0.008326	0.090582	0.059628	0.142893	0.004774	0.919080
ALIC_L	0.007628	0.004352	**0.000514**	0.006086	0.092391	0.007198	0.026067	0.005456	0.441977
ALIC_R	0.005968	0.003833	0.006318	0.001356	0.093413	0.018827	0.090946	**0.000891**	0.535277
BCC	0.013034	**0.000215**	0.001472	0.003271	0.079671	0.001340	0.004097	0.045797	0.226531
CGC_L	0.072926	0.051894	0.017171	0.165443	0.583171	0.030280	0.030024	0.133603	0.408166
CGC_R	0.860287	0.547693	0.135066	0.411077	0.614490	0.163572	0.368936	0.419927	0.178500
CGH_L	0.618555	0.042652	0.802660	0.506437	0.048936	0.364837	0.745948	0.810020	0.128463
CGH_R	0.752315	0.091256	0.613257	0.283103	0.007986	0.442902	0.829982	0.688043	0.311122
CR_L	0.035115	**0.000425**	0.001443	0.002692	0.132577	0.006525	0.033304	0.012142	0.333241
CR_R	0.019666	**0.000767**	**0.001050**	0.002786	0.051196	0.030318	0.069854	0.001295	0.264890
CST_L	0.788316	0.031726	0.563371	0.804118	0.565572	0.363281	0.391397	0.576812	0.312258
CST_R	0.500754	0.201734	0.433556	0.900428	0.881754	0.521319	0.767959	0.367192	0.414091
EC_L	0.085005	0.044982	0.019729	0.163228	0.726583	0.056892	0.055734	0.031084	0.988365
EC_R	0.094213	0.198775	0.075817	0.129711	0.558491	0.147070	0.187275	0.085525	0.694013
FX	0.925955	0.249383	0.212758	0.378159	0.103105	0.666299	0.805293	0.886774	0.373812
FX/ST_L	0.018626	0.012440	0.007294	0.007238	0.204365	0.198527	0.089628	**0.000834**	0.743805
FX/ST_R	0.041598	0.011069	0.003752	0.012061	0.245766	0.019039	0.054630	0.017545	0.471900
GCC	0.287623	0.064903	0.002816	0.168512	0.551797	0.018886	0.005231	0.239644	0.654170
IC_L	0.006070	0.004685	**0.000410**	0.006161	0.290737	0.006933	0.014592	0.005755	0.350488
IC_R	0.012827	0.009656	0.005557	0.001732	0.170797	0.033328	0.102842	0.003094	0.380861
IFO_L	0.070383	0.069665	0.002614	0.055351	0.288121	0.020055	0.001564	0.083841	0.093857
IFO_R	0.874375	0.100590	0.414204	0.292815	0.871867	0.114385	0.083871	0.511066	0.772739
PCR_L	0.052258	**0.000291**	**0.000338**	0.012555	0.492634	0.006169	0.039157	0.032150	0.148636
PCR_R	0.004682	0.002274	0.002477	0.004827	0.180613	0.012462	0.031396	0.006045	0.073790
PLIC_L	0.068801	0.031244	0.029781	0.039532	0.808580	0.165358	0.185160	0.216486	0.734145
PLIC_R	0.157330	0.088411	0.131976	0.022771	0.404028	0.292330	0.545357	0.008599	0.360206
PTR_L	0.028305	0.003246	0.002796	0.020879	0.358099	0.107905	0.193666	0.024496	0.320120
PTR_R	0.147446	0.124119	0.006914	0.078140	0.550166	0.646249	0.671288	0.093667	0.749256
RLIC_L	0.015647	0.038856	0.001717	0.044665	0.527763	0.010899	0.009799	0.004656	0.206317

RLIC_R	0.092438	0.119147	0.008252	0.033449	0.547880	0.103960	0.098317	0.077741	0.384912
SCC	0.028938	0.002908	0.003713	**0.000289**	0.011640	0.011191	0.119548	0.008324	0.075289
SCR_L	0.033189	**0.000275**	**0.000814**	**0.001009**	0.188472	0.020628	0.066296	0.018932	0.362265
SCR_R	0.013715	**0.000903**	**0.000883**	0.005811	0.034102	0.075549	0.120177	0.001661	0.157761
SFO_L	0.002775	0.008694	0.004399	0.008192	0.069469	0.023932	0.058337	0.005699	0.085714
SFO_R	0.083809	0.011136	0.022410	0.035749	0.263633	0.128138	0.314530	0.027016	0.198518
SLF_L	0.013409	**0.000790**	**0.000351**	0.011793	0.289282	0.005244	0.003482	0.021742	0.204972
SLF_R	0.010305	0.011133	0.028103	0.019198	0.199848	0.056144	0.083824	0.009703	0.151763
SS_L	0.195017	0.066968	0.012118	0.032182	0.190180	0.146178	0.212975	0.051752	0.950228
SS_R	0.049725	0.213823	0.067182	0.153005	0.973074	0.742326	0.353633	0.152876	0.700997
UNC_L	0.079325	0.005068	0.010309	0.016936	0.877875	0.003811	0.025114	0.021537	0.090164
UNC_R	0.062176	0.002434	0.029421	0.011474	0.717128	0.050724	0.130772	0.014999	0.318066

Partial correlation analysis between tract-based MD and different cognitive scores after correcting for age, sex, education and vascular risk factors. Abbreviations: MoCA, Montreal Cognitive Assessment; TMT-B, Trail-Making Tests B; SCWT-C, Stroop color-word test C; DSS, digital span substitution test; VFT, verbal fluency test; AVLT-4/5, auditory verbal learning test short and long delayed free recall; Rey-O copy, Rey-Osterrieth Complex Figure Test (copy), BNT, Boston Naming Test. Bold p values are statistically significant after Bonferroni correction.

Supplementary Figure 1 Group comparison results of ROI analysis for 42 tracts with statistical information

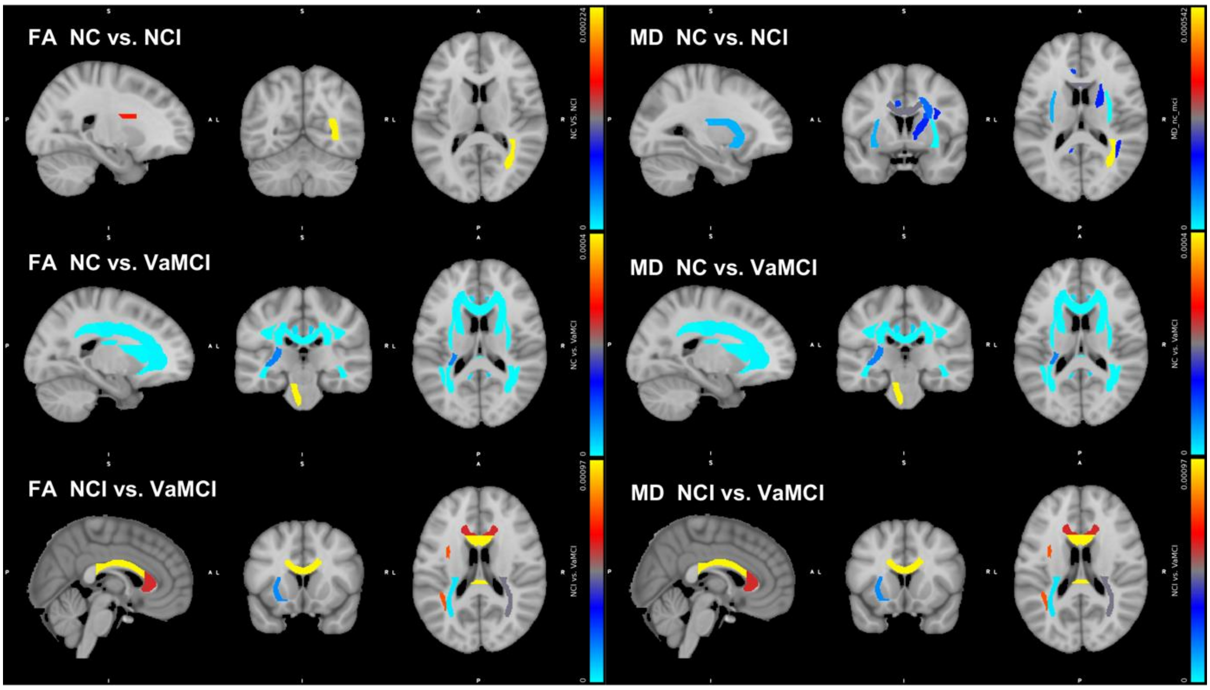

Supplementary Figure 1 *Group comparison of FA and MD on regional white matter tracts with the colours indicating the strength of p values. Left panels showed the FA difference between every two groups, Right panels showed the MD results between every two groups. Abbreviations: FA, skeletonized fractional anisotropy; MD, skeletonized mean diffusivity, VaMCI, CSVD with mild cognitive impairment; NCI, CSVD with no cognitive impairment; NC, normal control.*
